# High DNA methylation age deceleration defines an aggressive phenotype with immunoexclusion environments in endometrial carcinoma

**DOI:** 10.3389/fimmu.2023.1208223

**Published:** 2023-06-14

**Authors:** Jing Hao, Tiantian Liu, Yuchen Xiu, Huiyang Yuan, Dawei Xu

**Affiliations:** ^1^ Department of Clinical Laboratory, Qilu Hospital of Shandong University, Jinan, China; ^2^ Shandong Engineering Research Center of Biomarker and Artificial Intelligence Application, Jinan, China; ^3^ Department of Pathology, School of Basic Medical Sciences, Cheeloo College of Medicine, Shandong University, Jinan, China; ^4^ Department of Urology, Qilu Hospital of Shandong University, Jinan, China; ^5^ Department of Medicine, Bioclinicum and Center for Molecular Medicine (CMM), Karolinska Institutet and Karolinska University Hospital Solna, Stockholm, Sweden

**Keywords:** DNA methylation age, endometrial carcinoma, immunoexclusion, tumor microenvironment, prognosis, telomere maintenance

## Abstract

Like telomere shortening, global DNA hypomethylation occurs progressively with cellular divisions or *in vivo* aging and functions as a mitotic clock to restrain malignant transformation/progression. Several DNA-methylation (DNAm) age clocks have been established to precisely predict chronological age using normal tissues, but show DNAm age drift in tumors, which suggests disruption of this mitotic clock during carcinogenesis. Little is known about DNAm age alterations and biological/clinical implications in endometrial cancer (EC). Here we address these issues by analyzing TCGA and GSE67116 cohorts of ECs. Horvath clock analysis of these tumors unexpectedly revealed that almost 90% of them exhibited DNAm age deceleration (DNAmad) compared to patient chronological age. Combined with an additional clock named Phenoage, we identified a subset of tumors (82/429) with high DNAmad (hDNAmad+) as assessed by both clocks. Clinically, hDNAmad+ tumors were associated with advanced diseases and shorter patient survival, compared to hDNAmad- ones. Genetically, hDNAmad+ tumors were characterized by higher copy number alterations (CNAs) whereas lower tumor mutation burden. Functionally, hDNAmad+ tumors were enriched with cell cycle and DNA mismatch repair pathways. Increased PIK3CA alterations and downregulation of SCGB2A1, the inhibitor of PI3K kinase, in hDNAmad+ tumors, might promote tumor growth/proliferation and stemness. In addition, the inactivation of aging drivers/tumor suppressors (TP53, RB1, and CDKN2A) while enhanced telomere maintenance occurred more frequently in hDNAmad+ tumors, which supports sustained tumor growth. Prominently, hDNAmad+ tumors were featured with immunoexclusion microenvironments, accompanied by significantly higher levels of VTCN1 expression while lower PD-L1 and CTLA4 expression, which indicates their poor response to immune checkpoint inhibitor (ICI)-based immunotherapy. We further showed significantly higher levels of DNMT3A and 3B expression in hDNAmad+ than in hDNAmad- tumors. Thus, the tumor suppressive function of aging-like DNA hypomethylation is severely impaired in hDNAmad+ tumors, likely due to enhanced expression of DNMT3A/3B and dysregulated aging regulators. Our findings not only enrich biological knowledge of EC pathogenesis but also help improve EC risk stratification and precision ICI immunotherapy.

## Introduction

Endometrial carcinoma (EC) is among the most common malignancies of the female reproductive organs worldwide (1–3). The overall incidence of EC has doubled in the last two decades, and in 2020, approximately 420 000 new cases were diagnosed (1–3). Based on their pathogenic mechanisms, ECs are largely classified into two types. Type I, accounting for the majority of ECs, presumably results from the hyper-activity of estrogen signaling (1, 3, 4). The patients usually have low-grade carcinomas with favorable outcomes, however, a subset of them may progress into aggressive diseases (1). Type II ECs (up to 20% of all ECs) are in general estrogen-unrelated and high-grade with poor prognosis (1, 3). Therefore, for type II and progressive type I ECs, it is clinically important to identify reliable outcome predictors for patient stratification, and in that case, high-risk patients may be pinpointed for active surveillance and personalized intervention, which should be crucial to reducing disease-associated morbidity and mortality. Toward this end, many clinical and pathological variables have long been applied (1); moreover, molecular classifications of ECs were recently established for prognosis (5, 6). However, currently-existing predictors are far from sufficient to fully prognosticate patient outcomes, and therefore, further developments of more reliable biomarkers are required to achieve precision medicine of ECs.

Traditionally, surgery is the standard treatment for ECs, while patients at advanced stages or with recurrent/metastatic diseases also need adjuvant chemotherapy and/or radiotherapy (1). During the last decade, cancer immunotherapy by targeting immune checkpoint proteins (immune checkpoint inhibition, ICI) has been applied for various human malignancies including EC, which has revolutionized the cancer therapeutic landscape by demonstrating exceptional efficacy in various cancer types (1, 7); however, such therapeutic benefits are only observed in subsets of patients (7). Cancer microenvironments (CMEs) are a key factor to affect patient response to ICIs, for instance, lack of CD8 T cell infiltration, or so-called immunoexclusion phenotype, usually leads to ICI treatment failure (7, 8). Many intrinsic and external mechanisms participate in the regulation of immune cell infiltration and in ECs, DNA mismatch repair (dMMR) deficiency is known to increase CD8 T cell numbers in tumor tissues, whereas proficient dMMR-carrying tumors frequently exhibit an immunoexclusion feature (7, 9, 10). It has been shown that patients with dMMR-deficient tumors respond to ICIs (7, 10). However, the majority of EC tumors are dMMR proficient. Thus, there remain great need for profound mechanistic insights into tumor-immune interactions, thereby identifying novel predictive biomarkers to distinguish between ICI responders and non-responders.

DNA methylation, the methyl group-modified DNA, plays an essential role in the epigenetic regulation of gene transcription/expression, thereby participating in numerous physiological and pathological processes (11). Recent studies have revealed that DNA methylation alters in an age-dependent manner (12, 13), and in 2013, Horvath identified that 353 CpGs, differentially methylated during aging, served as a robust predictor for chronological age in multiple human tissues/organs, which was so-called Horvath clock (14). More recently, the next generation DNAm age clock, DNAm Phenoage (15), was further developed and observed to outperform the Horvath clock in predicting aging outcomes, including all-cause mortality, cancers, health-span, or physical functioning (15). In addition, several similar models were developed by others for largely the same purposes (12). Certain environmental factors or lifestyles and chronic conditions (such as diabetes and hypertension) have been shown to affect age-related DNA methylation, thereby leading to DNAm age acceleration or deceleration (DNAmaa or DNAmad) (16–20). DNAm age is generally in accordance with “biological” age (12). Consistently, DNAmaa has been observed to reliably predict age-related morbidity and mortality (12), and to promote cancer development (21).

Aging and cancer have been shown to share specific epigenomic alterations, and therefore the link between DNAm age and carcinogenesis has been explored (17, 22, 23). Both blood- and tissue-based analyses suggest that DNAmaa increases cancer susceptibility, while DNAmaa in tumors predicts poor patient outcomes in different types of cancer (24–33). However, by mapping DNA methylation, chromatin, and genome topological landscapes in colorectal cancer (CRC) and normal colon tissues, Johnstone et al. (34) demonstrated that age-related global hypomethylation led to chromatin reconfigurations, thereby downregulating expression of protein-coding genes, especially those promoting stem cell proliferation, epithelial-mesenchymal transition (EMT), and invasion. Thus, global hypomethylation is suggested as a tumor suppression mechanism, and overcoming it is thus required for cancer development and/or progression (22). From this point of view, DNAmad may play a more important role in promoting cancer aggressiveness.

Age-related global DNA hypomethylation (coupled with focal hypermethylation of specific CpG islands) occurs in cells having undergone many times doubling and is much more prominent in proliferative epithelial tissues *in vivo* (22, 34). A woman’s endometrium experiences up to 450 cycles of proliferation and regeneration throughout her reproductive lifespan (35), and analyses of methylation changes in endometrium-derived EC may be more informative. However, little has been known about the DNAm age alterations in ECs. In the present study, we determined whether DNAmaa and DNAmad were involved in the EC formation/progression and had clinical implications in the disease prognosis by analyzing the TCGA (5) and GSE67116 (36) cohorts of EC patients. We show that DNAmad in tumors is widespread and high DNAmad (hDNAmad) stratifies the aggressive tumor phenotype with immunoexclusion microenvironments in ECs.

## Materials and methods

### Study subjects/specimens, clinico-pathological, DNA methylation, and expression data processing

The Cancer Genome Atlas (TCGA) cohort of EC includes 432 patients with 432 tumors and 46 non-tumorous endometrial (NTE) specimens (5). Clinical and pathological information, DNA methylation (Illumina 450K platform) data, mutation, and copy number data were downloaded from Feb. 2022. RNA sequencing results of those tumors were also downloaded simultaneously, and mRNA abundances were expressed as RSEM (RNA-Seq by Expectation Maximization).

The GSE67116 cohort of patients includes 8 hyperplasia, 33 primary, and 53 metastatic EC tumors, and specimens from these patients were analyzed for DNA methylation profiling using Illumina HumanMethylation450 BeadChip (36). The methylation data were downloaded in Feb. 2022.

### Calculation for DNAm age, DNAm age acceleration, and deceleration

The Horvath clock was developed to predict chronological age by assessing the methylation statuses of 353 CpGs in 2013 (14). These 353 CpGs are selected based on a penalized regression evaluation, and with age increase, 190 of them get hypermethylated while 160 are hypomethylated. DNAm age was thus calculated according to the methylation beta values of 353 CpGs using the following formula (https://dnamage.genetics.ucla.edu).


$$\text{DNAmAge}=\text{inverse}:\text{F}({\text{α}}_{0}+{\text{α}}_{1}{\text{CpG}}_{1}+\ldots +{\text{α}}_{353}{\text{CpG}}_{353}).$$

where F is a function for the transformation of age and α_i_s are coefficients generated from the elastic net regression model. The calculation accuracy was evaluated using the mean absolute difference (MAD) between DNAm and chronological age. DNAmaa or DNAmad is simply expressed as a deviation between chronological age and DNAm age, or residual of DNAm age extracted from chronological age.

The phenoage clock was introduced in 2018 for chronological age prediction based on 513 CpGs (37). Forty-one of these 513 CpGs overlap with those in the Horvath clock. The DNAm age calculation using this clock is principally as same as above with a formula:


$$\text{DNAm PhenoAge}=\text{intercept}+{\text{CpG}}_{1}\times \text{β}1+\ldots +{\text{CpG}}_{513}\times \text{β}513$$

### Identification of differentially methylated probes

For differences in global DNA methylation among different groups, DMPs were sorted out using |Δβ|> 0.20 and adjusted *P*<0.05. Those age-related DMPs in Horvath and Phenoage clocks were excluded. The volcano plot of DMPs was made and visualized with the R package “ggplot2”.

### Differentially expressed gene analysis

RNA sequencing-based gene expression abundances were determined using Transcripts Per Kilobase Million (TPM) and log2(x + 1) transformed. DEGs between tumors with and without hDNAmad were identified using edgeR packages in R software. An adjusted p < 0.05 and fold change(log2) > 1.5 were considered statistically significant.

### Kyoto Encyclopedia of Genes and Genomes enrichment analyses and Gene Set enrichment analysis

Reference gene signatures for Kyoto Encyclopedia of Genes and Genomes (KEGG) analysis were downloaded from https://www.gsea-msigdb.org/gsea/index.jsp (h.all.v2022.1.Hs.symbols.gmt’ and ‘c2.cp.kegg.v2022.1.Hs.symbols.gmt’). Differences in KEGG pathways between two DNAm age groups were determined using GSEA (version 4.2.1). Adjusted *P* value <0.05 and *FDR <*0.25 were regarded as significantly different pathways. Heatmap was made using the R package “ComplexHeatmap”.

### Copy number alteration, aneuploidy score, and tumor mutation burden analysis

Somatic CNAs were downloaded from https://xenabrowser.net/. CNA plots were made using the R package ‘oncoPrint’ in ‘ComplexHeatmap’. Aneuploidy scores were the sum total of altered (amplified or deleted) chromosome arms. TMB is defined as the number of non-silent mutations per million bases and the data were downloaded from https://xenabrowser.net/.

### Analyses of immune cell infiltration and immune checkpoint factor expression

The CIBERSORT algorithm was used to determine the proportion of the 22 types of immune cells in each EC tumor based on RNA sequencing data (38). The calculation was conducted online at http://cibersort.stanford.edu. We also calculated myeloid and Teffector signatures to estimate tumor infiltrated myeloid and CD8 T cells using single sample GSEA (ssGSEA), and their signature gene panels were: myeloid signature: IL6, CXCL1, CXCL2, CXCL3, CXCL8, and PTGS2; Teffector signature: CD8A, EOMES, PRF1, IFNG, and CD274. Immune checkpoint factor analyses were carried out as previously described (39). Cancer immune cycle analysis was performed based on Xu et al. at the website https://github.com/dengchunyu/TIP (40).

### Analyses for proliferation, cell cycle score, stemness, and telomerase score

EC tumor proliferation was evaluated using expression levels of Ki-67 mRNA and cell cycle scores, respectively. Cell cycle score was calculated based on single sample GSEA (ssGSEA) using the following gene panel: CDK2, CDK4, CDK6, BUB1B, CCNE1, POLQ, AURKA, KI-67, and CCNB2. The stemness score was calculated based on ssGSEA of 109 gene signatures as described (41). The telomerase score was calculated according to expression levels of 10 telomerase components (TERT, TERC, DKC1, TCAB1, NHP2, GAR1, NOP10, RUVBL1 and 2, and NVL) as described (42).

### Statistical analyses

Statistical analyses were performed using R package version 4.0.5 or PFSS. According to data distributions, Student’s t-test, Wilcox and K-W sum tests, and Chi^2^- or Fish exact tests were used for analysis. The correlation between DNAm age and chronological age was evaluated by Pearson coefficient correlation. Kaplan–Meier analysis with log-rank test was carried out to evaluate overall- and progression-free survivals (OS and PFS) among groups. The effect of various quantitative variables on OS and PFS was measured by univariate and multivariate Cox regression analyses. *P* < 0.05 were considered statistically significant.

## Results

### DNAm age and correlation with chronological age in NT specimens and EC tumors

**Figure 1:**
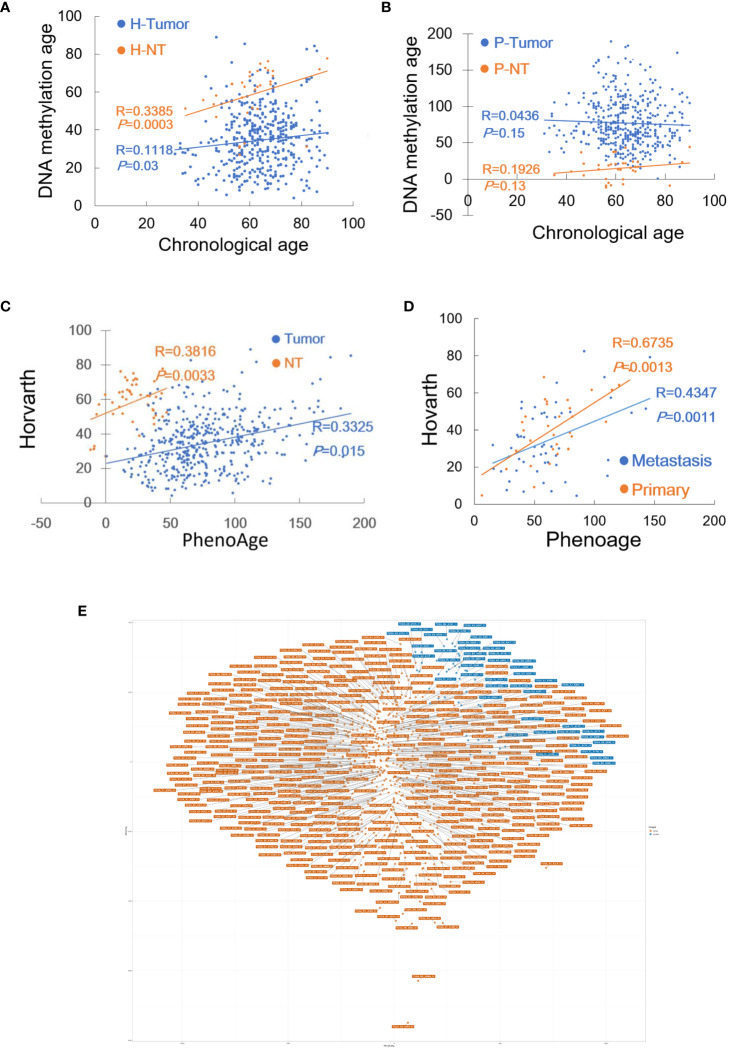
DNAm age in EC tumors as determined by Horvath and Phenoage clocks. Horvath and Phenoage clocks were used to calculate DNAm age in TCGA and GSE 67116 cohorts of EC tumors as described in Methods. **(A)** DNAm age in the TCGA EC cohort as calculated using the Horvath clock. Orange and blue dots: NTs and tumors, respectively. **(B)** DNAm age in the TCGA EC cohort as calculated using the Phenoage clock. Orange and blue dots: NTs and tumors, respectively. **(C)** The correlation of DNAm age as determined using Horvath and Phenoage clocks in the TCGA cohort of NTs and tumors. **(D)** The correlation of DNAm age as determined using Horvath and Phenoage clocks in the GSE67116 cohort of primary and metastasis EC tumors. Orange and blue dots: Primary and metastatic EC tumors, respectively. **(E)** Principal component analyses (PCA) of the high DNAmad-positive and negative tumors in the TCGA EC cohort.

In the TCGA cohort, patient age information was unavailable in 3 of 432 tumors and 12 of 46 NT specimens, and therefore the analysis was performed on 429 tumors and 32 NTs. We first employed the Horvath model to measure their DNAm age. DNAm age in the NTs showed a significant correlation with patient chronological age (Pearson correlation R = 0.339, *P* = 0.0003, and MAD = 9 yrs with range -51 - +16) (**Figure 1A**). The analyses of 429 EC tumors showed no correlation between DNAm and chronological ages in tumors (R = 0.112, *P* = 0.030) (**Figure 1A**), and their MAD was 12.0 yrs (range -79 – +42). Fourteen of these tumors had DNAm age largely as same as patient chronological age (difference < 3 yrs), while 12 exhibited DNAm age older than chronological age with a difference from 3 to 42 yrs or DNAm age acceleration (DNAmaa), and the remaining 403 had DNAm age younger than chronological age (-3 to -79 yrs) or DNAm age deceleration (DNAmad).

It was unexpected that more than 90% of EC tumors displayed DNAmad as assessed using the Horvath clock. Given such observations, the Phenoage clock was further applied for those same specimens. The phenoage-based analyses showed that neither NT nor tumor specimens had a correlation between chronological age and DNAm age (NTs, R = 0.193, *P* = 0.150) (Tumors, R = 0.044, *P* = 0.939, and MAD = 12 yrs with range -76 - +132) (**Figure 1B**). In 24 of 429 EC tumors, DNAm and patient chronological ages were largely matched with differences <3 yrs, whereas DNAmaa and DNAmad were identified in 251 and 152 of them, respectively.

Horvath and phenoage clock calculations gave rise to different results in the TCGA cohort EC analyses, however, there was a significant correlation between DNAm ages estimated by two models (**Figure 1C**). To validate these findings, we further measured DNAm age in the GSE67116 cohort of 86 EC patients. All patient age was 53 yrs old and there were 33 primary and 53 metastatic ECs plus 8 hyperplasias. For tumors from 86 patients, the Horvath clock calculation showed that 8 of them had largely matched DNAm and chronological age (difference within 3 yrs), while DNAmaa and DNAmad occurred in 14 and 64, respectively (MAD = 13 yrs, and range -48 to 30 yrs with median -17). The Phenoage clock was further applied for these tumors, and 52 of 86 tumors exhibited DNAmaa while 31 had DNAmad (MAD = 18 yrs, and range -47 to 181 yrs with median -7). DNAm ages calculated using two models were significantly correlated (For primary and metastatic ECs, R = 0.674, *P* = 0.001, and R = 0.435, *P* = 0.026, respectively) (**Figure 1D**).

Analyses of two EC cohorts unveiled widespread DNAm age disruption. Tumor DNAm ages as calculated using Horvath and Phenoage clocks were different, but they correlated with each other significantly. Because >90% of tumors in the TCGA cohort exhibited DNAmad as determined using the Horvath clock, we focused on tumors with DNAmad. We first selected the top 1/3 (134) DNAmad tumors and further examined their DNAm age using the Phenoage clock. A total of 82 tumors were identified to display DNAmad as determined using both models, which we named high DNAmad (hDNAmad) for all next analyses. Principal component analysis (PCA) of 82 hDNAmad and remaining EC tumors showed that they were largely segregated, although not completely (**Figure 1E**).

### Association of hDNAmad with clinic-pathological characteristics of EC patients

We next determined whether there is an association between 82 hDNAmad+ EC tumors and clinic-pathological variables. As listed in **Table S1**, hDNAmad occurred at significantly higher percentages in patients with age ≥60 yrs, serous or mixed histological types, and advanced stages and grades. In contrast, the hDNAmad frequency was significantly lower in patients with diabetes and hypertension (**Table S1**). Patient BMI had no impact on hDNAm age (**Table S1**).

### hDNAmad as a predictor for shorter survival in EC patients

**Figure 2:**
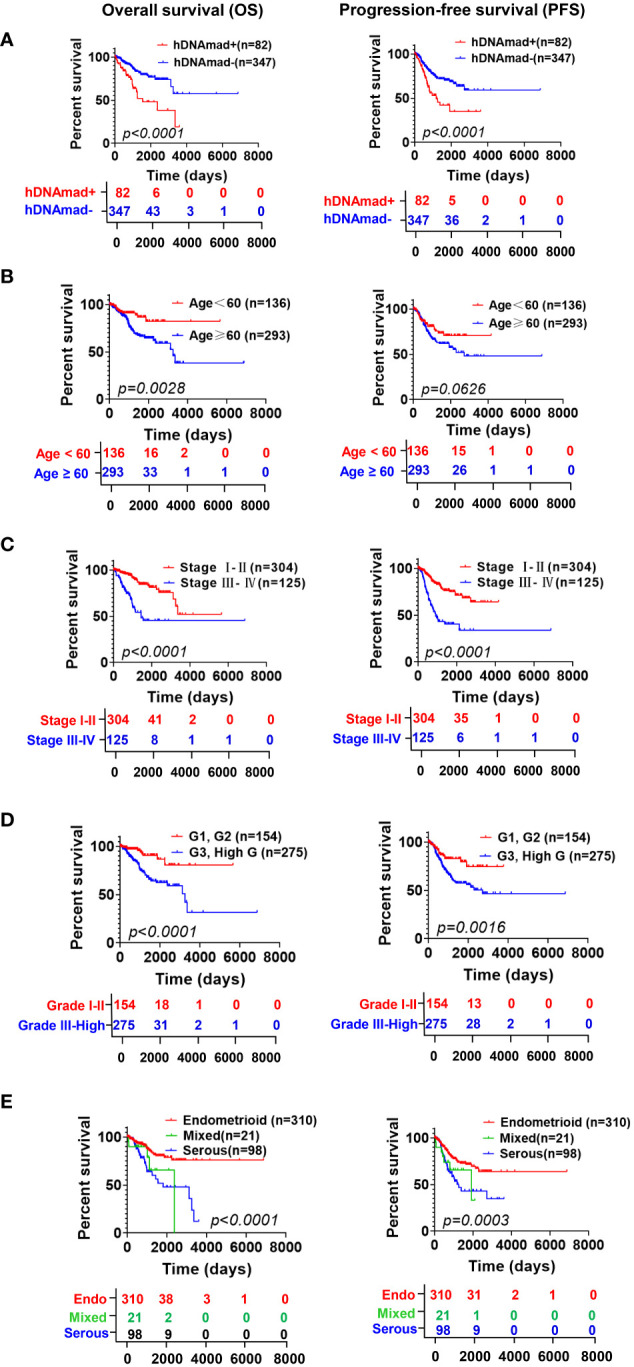
The impact of hDNAmad, and clinic-pathological variables on overall and progression-free survival (OS and PFS) in the TCGA cohort of EC patients. DNAm **(A)** and chronological age **(B)**, stage **(C)**, grade **(D)**, and histology **(E)** were included for Kaplan–Meier analysis with a log-rank test. A total of 429 patients were analyzed.

Both DNAmaa and DNAmad have been observed to be associated with survival in cancer-type-dependent manners. Thus, we sought to determine the impact of hDNAmad on patient OS and PFS. Kaplan-Meier analyses revealed that patients in the hDNAmad+ group had significantly shorter OS and PFS (**Figure 2**). In the meanwhile, we also evaluated the association between survival and clinic-pathological variables including chronological age (<60 vs ≥60 yrs), stages (I +II vs III + IV), grades (I +II vs III + high) and histological types (endometroid vs serous + mixed). As expected, senior ages, advanced stages/grades, and non-endometroid types all predicted significantly shortened OS and PFS (**Figure 2**).

Given these observations, we further performed univariate and multivariate Cox regression analyses to assess the impact of the above variables on OS and PFS. The result is summarized in **Table S2**. hDNAmad, age >60 yrs, advanced stages and grades, and non-endometroid histology all predicted significantly shorter OS in univariate analyses, whereas hDNAmad and advanced stages/grades remained significantly associated with OS in multivariate analyses. PFS was determined in the same manner (**Table S2**), and univariate analyses showed that only chronological age had no impact on PFS. Multivariate analyses revealed hDNAmad and advanced stages as independent prognostic factors for shortened PFS.

### hDNAmad association with EC molecular subtypes and genomic alterations

ECs have been molecularly classified into the following 4 subtypes with different outcomes: CN-high, CN-low, microsatellite instability (MSI) (hypermutated), and PLOE (ultramutated) (5). We further sought to assess the relationship of hDNAmad with molecular subtypes in ECs. Of 199 patients with the molecular subtype information available, 41 of them harbored hDNAmad+ tumors, and their frequencies were 43.9%, 4.9%, 19.5%, and 0% in CN-high, CN-low, SMI, and PLOE subtypes, respectively. The distribution of hDNAmad- tumors was 9.3%, 22.7%, 43.0%, and 4.2% in CN-high, CN-low, SMI, and PLOE subtypes, respectively. CN-high and MSI subtypes were highly prevalent in hDNAmad+ and – tumors, respectively (*P* < 0.0001).

**Figure 3:**
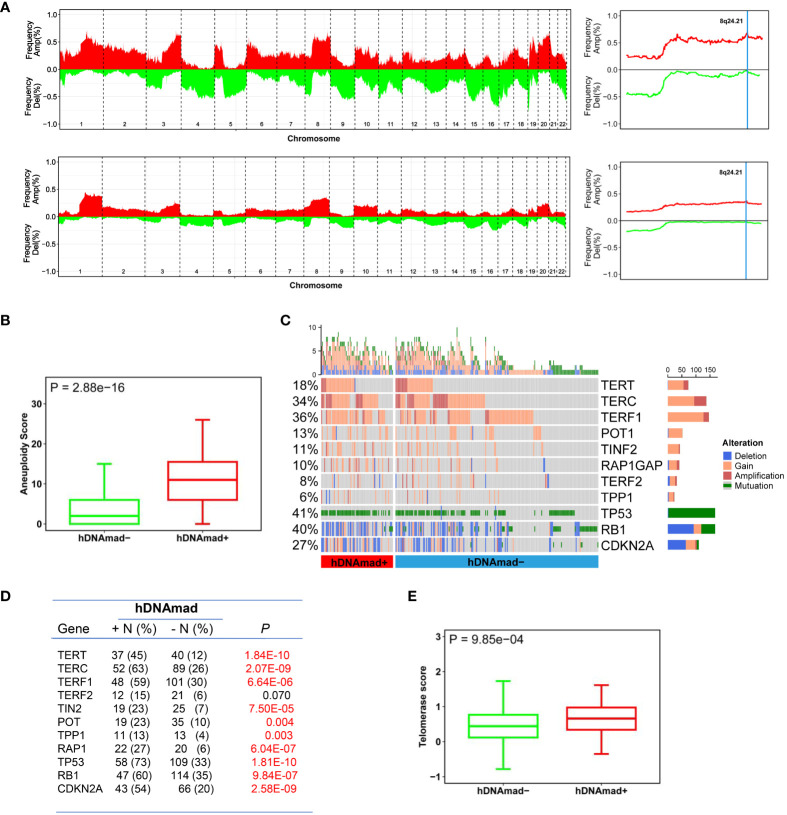
Differences in copy number alterations (CNAs) and telomerase activity between hDNAmad+ and – EC tumors. The TCGA cohort of 429 EC tumors was analyzed. **(A)** Comparison of global CNAs between two tumor groups. Left panels: Plots illustrating frequencies of gain/amplification (Red) and deletion (Green) in 22 chromosomes. Top and bottom: hDNAmad+ and – tumors, respectively. Right panels: The detailed analysis of chromosome 8q24.21 where the *MYC* gene is located. Top and bottom: hDNAmad+ and – tumors, respectively. **(B)** Aneuploidy scores, the sum total of altered (amplified or deleted) chromosome arms as shown in **(A)**, were calculated and compared between hDNAmad+ and – tumors. **(C, D)** CNAs in genes responsible for aging regulation and *TP53* gene mutations in EC tumors. Differences in CNAs and mutations between hDNAmad+ and – tumors were detailed in **(D)**. **(E)** Differences in telomerase activity between hDNAmad+ and – tumors. Telomerase activity was expressed as telomerase score and calculated based on expression levels of 10 telomerase components.

Given the above finding that most hDNAmad+ tumors were in the CN-high subtype, we further analyzed all 429 EC patients to determine global gene gain and deletion in tumors. Whole genome analyses revealed higher frequencies of CNAs (**Figure 3A**) and a robustly higher aneuploidy score (**Figure 3B**) in the hDNAmad+ tumors (hDNAmad+ vs -: *P* = 2.88E-16). Specific dissections of chromosome 8 showed that the hDNAmad+ tumors harbored markedly higher incidences of 8q24.1 region where the *MYC* gene is located (hDNAmad+ vs -: 61% vs 35%, *P* = 0.0005) (**Figure 3A** right).

We pay special attention to alterations in telomere maintenance genes due to the important role of telomerase and telomeres in cellular senescence and DNAm age regulation (12, 43). To this end, the copy numbers of the telomerase core holyenzyme subunits including telomerase reverse transcriptase (TERT) and telomerase RNA component (TERC), and six telomere-binding factors or shelterin proteins (TERF1, TERF2, POT1, TPP1, TINF2, and RAP1) were analyzed (**Figures 3C, D**). The *TERT* gain or amplification was frequent, with 45% and 12% in tumors with and without hDNAmad, respectively (*P* = 1.84E-10). *TERC* aberrations were even highly prevalent, occurring in 63% and 26% of the two groups, respectively (*P* = 2.07E-09). There were also significant differences in aberrations of 5 shelterin genes except TERF2 between the hDNAmad+ and - tumors. The gain/amplification of the *TERF1* gene occurred most frequently (a total of 35% in 429 EC tumors), and its incidences were 59% and 30% for hDNAmad+ and - tumors, respectively (*P* = 6.64E-06). For the other 4 shelterin factors (TINF2, RAP1, TPP1, and POT1), genomic alterations were all significantly higher in hDNAmad+ than hDNAmad- tumors. Finally, we calculated telomerase scores using expression levels of 10 telomerase components. A significantly higher telomerase score was observed in hDNAmad+ than in hDNAmad- tumors (*P* = 6.64E-06) (**Figure 3E**).

In addition, because tumor suppressors TP53, RB1, and CDKN2A significantly contribute to aging regulation, we specifically examined potential differences between two groups of EC tumors, too (**Figures 3C, D**). The *TP53* mutation occurred in 57 of 82 (70%) and 105 of 347 (30%) hDNAmad+ and -tumors, respectively, and there was a highly significant difference (*P* < 0.0001). Similar differences in RB1 and CDKN2A alterations were observed (**Figures 3C, D**).

### Identification of DEGs and enriched pathways in hDNAmad+ EC tumors

**Figure 4:**
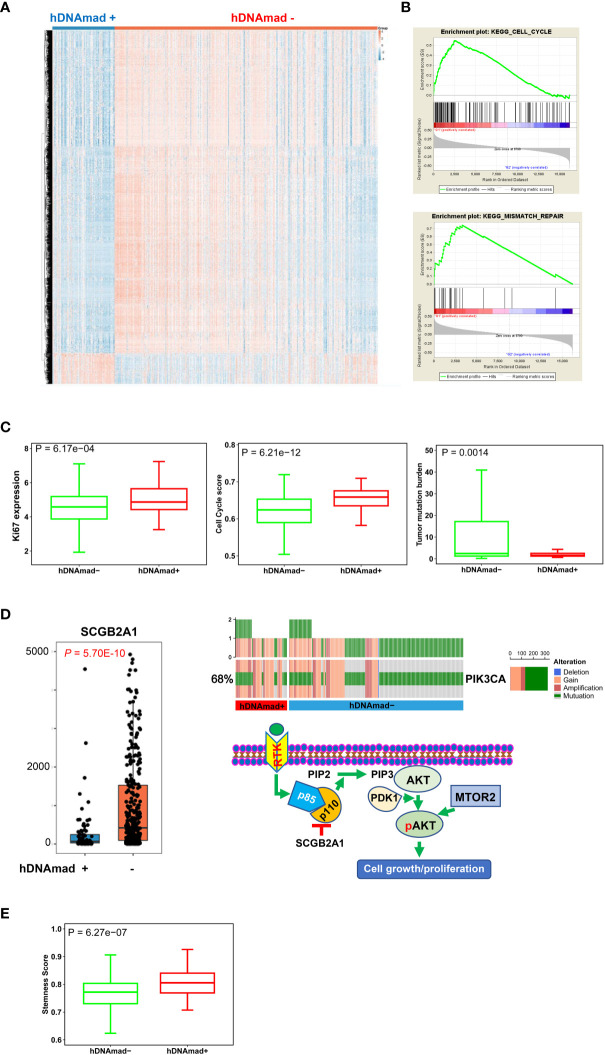
Differentially expressed genes (DEGs) and pathway enrichments between hDNAmad+ and – tumors in the TCGA EC cohort. A total of 429 tumors were analyzed. **(A)** The heatmap showing DEGs between hDNAmad+ and – EC tumors. **(B)** The identification of enriched cell cycle and DNA mismatch repair pathways in hDNAmad+ tumors by GSEA analysis. **(C)** Significantly higher expression of Ki-67 and cell cycle score in hDNAmad+ tumors. **(D)** The hyperactivation of the PI3K-AKT pathway in hDNAmad+ tumors. Left: Diminished SCGB2A expression in hDNAmad+ tumors. Right: Top plot showing increased PIK3CA amplification and mutations in hDNAmad+ tumors. The bottom schematic illustrates how increased PIK3CA alterations and downregulated SCGB2A1 result in PI3K-AKT pathway activation, thereby promoting cell proliferation. In hDNAmad+ tumors, higher frequency of *PIK3CA* [encoding p110α, the phosphatidylinositol 3-kinase (PI3K) subunit] amplification and mutation enhances PI3K activity, while lower expression of SCGB2A1, an inhibitor of PIK3CA kinase, further augments PI3K signaling activation. Such hyperactivated PI3K proteins catalyze more phosphorylation of PIP2 to generate PIP3. PDK1 then phosphorylates AKT in the presence of PIP3, which, together with MTOR2-mediated phosphorylation, consequently, activates AKT. The super-activation of the PI3K-AKT cascade promotes EC cell proliferation. RTK: Receptor tyrosine kinase; PIP2, phosphatidylinositol 4,5-bisphosphate; PIP3, phosphatidylinositol 3,4,5-trisphosphate. **(E)** Enhanced stemness score in hDNAmad+ EC tumors.

We then sought to determine DEGs and pathway alterations between hDNAmad+ and - tumors. A total of 693 DEGs were identified, and 269 of them were upregulated whereas 424 were downregulated in the hDNAmad+ tumors (**Figure 4A**, **Table S3**). GSEA analysis for KEGG pathways was then performed based on those DEGs. Cell cycle and mismatch repair pathways were significantly overrepresented within hDNAmad+ tumors (**Figure 4B**). To validate the GSEA analysis results, we further conducted comparisons between two groups by analyzing Ki-67 expression, a specific proliferation biomarker, cell cycle score, and tumor mutation burden (TMB). As shown in **Figure 4C**, hDNAmad+ tumors displayed significantly higher levels of Ki-67 and cell cycle scores while lower TMB than did hDNAmad- tumors (*P* = 6.17E-04, 6.21E-12 and 0.0014 for Ki-67, cell cycle score and TMB, respectively).

We also noticed that the secretoglobin (SCGB) family member SCGB2A1 was on the top of the DEG list with almost 5-fold higher expression in hDNAmad- than + tumors (**Figure 4D**). SCGB2A1 was recently identified as a negative regulator in the PI3K-AKT pathway (**Figure 4D**) (44), and to further evaluate this oncogenic signaling, we analyzed *PIK3CA* alterations. The total frequency of *PIK3CA* gain/amplification and mutation was 80% and 65% for hDNAmad+ and – tumors, respectively (*P* = 0.0004) (**Figure 4D**). In addition, as SCGB2A1 is preferably expressed in differentiated endometrial cells and differentiated EC tumors (45), we examined cancer stem cell phenotype. Consistently, the stemness score was notably higher in hDNAmad+ tumors (*P* = 6.27E-07) (**Figure 4E**).

### Immunoexclusion microenvironments in hDNAmad+ EC tumors

**Figure 5:**
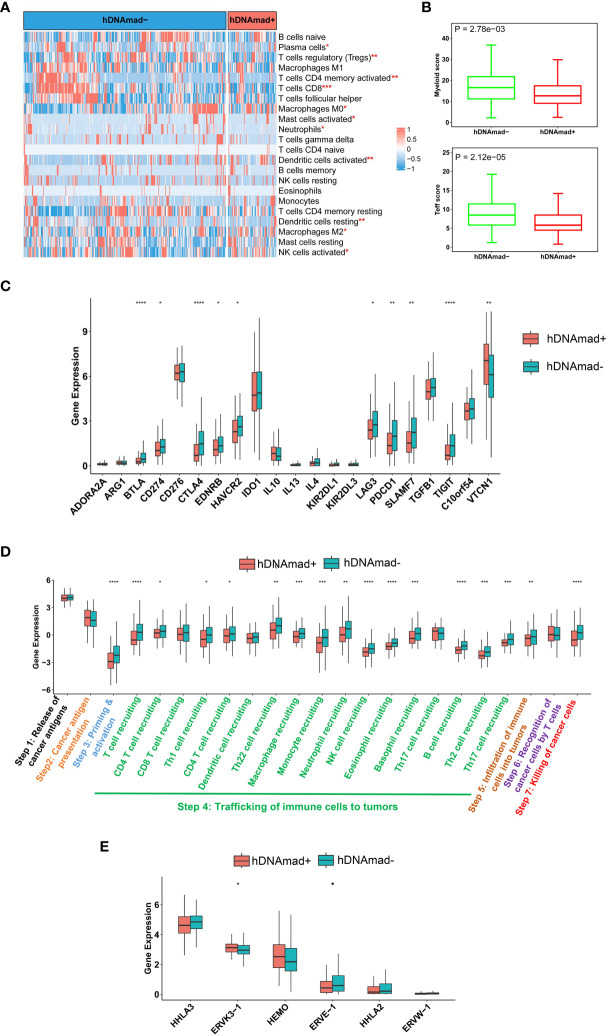
Identification of immunoexclusion microenvironments in hDNAmad+ tumors. A total of 429 tumors in the TCGA EC cohort were analyzed. **(A)** The heatmap shows reduced infiltrations of immune cells in hDNAmad+ tumors, as determined using the CIBERSORT analysis. *, **, and ***: P<0.05, 0.01, and 0.001, respectively. **(B)** Significantly lower myeloid and T effector scores in hDNAmad+ tumors. **(C)** Immune checkpoint factor expression analyses. **(D)** Cancer immune cycle analysis. ****, P<0.0001. **(E)** Human endogenous retrovirus (HERV) gene expression in hDNAmad+ and hDNAmad– tumors. Expression of 7 HERVs from the TCGA EC tumors was analyzed and compared between hDNAmad+ and hDNAmad– tumors. *, P<0.05.

We performed the CIBERSORT analysis to calculate the proportions of 22 kinds of infiltrated immune cells (38). As shown in **Figure 5A**, the most significant difference was the reduced CD8 T cells coupled with lower myeloid cells in hDNAmad+ tumors. To validate the findings above, we further evaluated myeloid and Teff cell signatures and similar results were obtained: Both myeloid and Teff signature scores were significantly lower in hDNAmad+ than - tumors (**Figure 5B**). Expression of 21 immune checkpoint genes was also compared, and 10 of them exhibited differential levels between two tumor groups (**Figure 5C**). VTCN1 (B7-H4) was the only upregulated gene, while CD274 (PD-L1), CTLA4, TIGIT, PDCD1, BTLA4, LAG3, EDNRB, HAVCR2, and SLAMF1 were all downregulated in hDNAmad+ tumors (**Figure 5C**).

A 7-step cancer immune cycle has been suggested, which includes the release of cancer cell antigens (step 1), cancer antigen presentation (step 2), priming and activation (step 3), trafficking of immune cells to tumors (step 4), infiltration of immune cells into tumors (step 5), recognition of cancer cells by T cells (step 6), and killing of cancer cells (step7) (40). To further dissect the impaired activity of anticancer immunity in hDNAmad+ tumors, we performed the tracking tumor immunophenotype (TIP) analysis (40). As shown in **Figure 5D**, the major defect in hDNAmad+ tumors was the trafficking of immune cells into tumor tissues, and the recruitment of most immune cell types (14/17) was significantly reduced compared to hDNAmad- tumors. These results are largely consistent with the CIBERSORT analysis. The diminished immune cell recruitment consequently led to declines in infiltrated cells and tumor killing (**Figure 5D**).

Johnstone et al. recently showed that aging-like global hypomethylation in cancer induces de-repression of *human endogenous retrovirus* (*HERV*) genes, thereby provoking anti-tumor immunity (34). We thus compared differences in HERV expression. Analyses of 7 HERVs showed significantly higher ERVK3-1 while lower ERVE-1 expression in hDNAmad+ tumors than in hDNAmad– ones (**Figure 5E**). The expression of the remaining 4 HERVs did not differ between the two tumor groups (**Figure 5E**), whereas HHLA-1 transcripts were undetectable in the vast majority of tumors (both groups) (data not shown).

### Differences in global DNA methylation and DNMT expression between hDNAmad+ and - EC tumors

**Figure 6:**
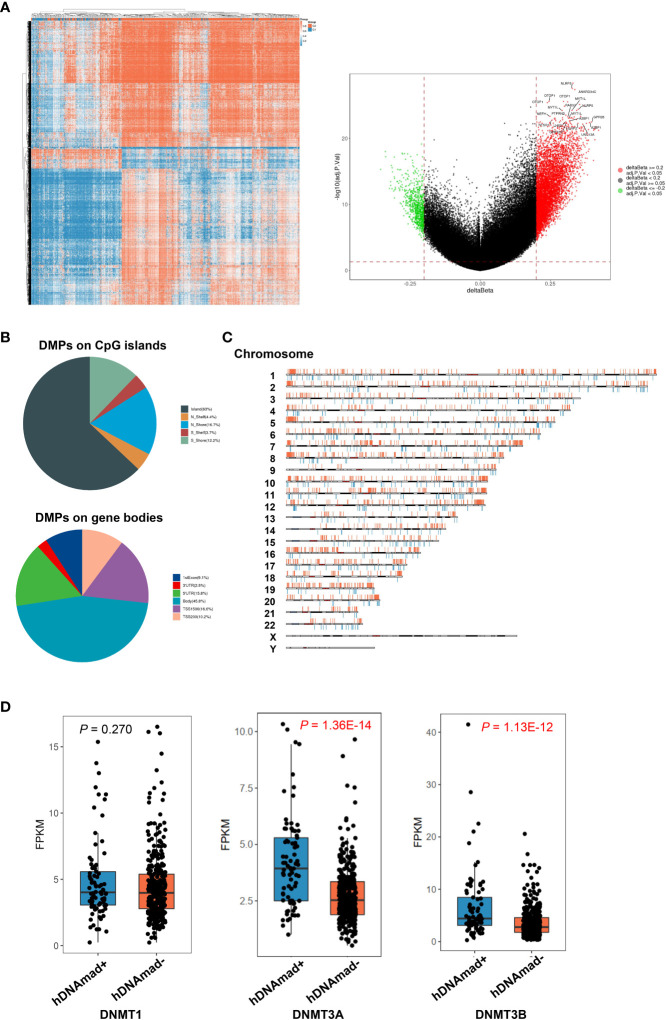
Differentially methylated probes (DMPs) and DNMT expression between hDNAmad+ and – tumors in the TCGA EC cohort. A total of 429 tumors were analyzed. **(A)** The heatmap (left) and volcano plot (right) illustrate DMPs between hDNAmad+ and – tumors. **(B)** Pie charts showing the percentage of hypomethylated and hypermethylated probes across different genomic regions (CpG islands: Shore probes located less than 2 kb from CpG island, Shelf = probes located >2 k from CpG islands and Gene Poor Region = probes not in island or annotated genes) and gene body regions [TSS1500 and TSS200—probes located within 1500 and 200 bp from TSS, respectively; 5′ untranslated regions (UTR); first exon; Body and 3′UTR]. **(C)** The distribution of DMPs on chromosomes. **(D)** Differences in expression of DNMAT1, DNMAT3A, and 3B between hDNAmad+ and – tumors.

Both Horvath and Phenoage clocks estimate DNAm age based on age-related alterations in DNA methylation, and we further sought to determine differences in global DNA methylation between hDNAmad+ and – EC tumors. A total of 6 953 differential methylation probes (DMPs) were identified between these two groups. The vast majority of DMPs (6 184/6 953, 89%) were hypomethylated while 769 of them (11%) were hypermethylated in hDNAmad+ tumors (**Figure 6A**). NLRP5, ANKRD34C, OTOP1, MYT1L, RAB37, NEFH, PTPRN2, GPR26, GPR123, NTSR1, LHX3, LILRP2, and UNG13A were the most differentially methylated genes between two groups (**Figure 6B**). Most DMPs were in CpG islands (CpGi) and gene body regions (**Figure 6C**). In addition, these DMPs were in general evenly distributed across 22 chromosomes (**Figure 6D**).

The DNMT family members DNMT1, DNMT3A, and 3B are essential to maintain DNA methylation, while their downregulation in senescent human cells contributes to global hypomethylation. Thus, we compared their expression. There was no difference in DNMT1 mRNA levels between the two groups, while DNMT3A and 3B expression were significantly upregulated in the hDNAmad+ tumors (*P* = 1.36E-14 and 1.13E-12, respectively) (**Figure 6D**).

## Discussion

Horvath and DNAm Phenoage clocks have been widely applied to estimate DNAm age in various tissue types, both normal and tumorous. In tissues from healthy individuals, DNAm age is highly correlated with chronological age, however, significant DNAmaa may occur in individuals who are smokers, obese, or have other unhealthy lifestyles (16–20). Several lines of evidence have suggested that DNAmaa is strongly associated with an increased risk of death from all-natural causes or serves as a predictor for life expectation (12, 14, 15, 46). In contrast, DNAmad is infrequently seen in healthy individual-derived tissues. Unlike normal tissues, however, tumors often exhibit high degrees of DNAm age drift where DNAmaa is predominant, although DNAmad occurs at low frequencies. Dependent on cancer type, DNAmaa and DNAmad may be associated with either worse or better patient outcomes. In the present study, we measured DNAm age in EC tumors using both Horvath and Phenoage clocks. DNAm age as determined by these two models was highly correlated with each other and most tumors exhibited strong age deacceleration or DNAmad compared to patient chronological age. However, differences were observed between the results obtained from the two models. Likely, the Horvath clock reflects chronological age while the Phenoage model is more closely associated with phenotypic aging (15). Moreover, we further revealed that hDNAmad was associated with an aggressive phenotype and poor outcomes in ECs.

Global hypomethylation has been suggested as a “mitotic clock” that records replication times of somatic cells and occurs progressively with cellular aging due to imperfect maintenance of DNA methylation. Casillas et al. showed that expression of DNMT members DNMT1, DNMT3a, and 3b was downregulated in normal human cells at late passages or senescent stage (47), which contributes to impaired DNA methylation maintenance and hypomethylation. Unexpectedly, we observed a widespread DNAmad in EC tumors. These results suggest that hypomethylation may be overcome during EC pathogenesis. Indeed, we demonstrated upregulated expression of DNMT family genes in hDNAmad+ tumors, which provides further support. In addition, tumor suppressors TP53, RB1, or CDKN2A, and telomere maintenance mechanisms play essential roles in aging regulation, and intriguingly, hDNAmad+ EC tumors had robustly higher frequencies of inactivation of TP53, RB1, and CDKN2A accompanied by significantly enhanced telomerase activity. Likely, all the above alterations cooperate to result in the inversion of age-related DNA methylation profiles in ECs.

One of the key findings is the identification of immunoexclusion microenvironments in hDNAmad+ EC tumors. The survey of immune and inflammatory cells in EC tumors unraveled lower numbers of infiltrated CD8 T and myeloid cells as the most marked feature in hDNAmad+ tumors. Immune checkpoint gene analyses showed elevated VTCN1 expression coupled with lower levels of PD-L1 and CTLA4 in these tumors. Immune-cold tumor microenvironments are known to be characterized by low levels of PD-L1 and CTLA4 coupled with enhanced VTCN1 expression (48). Collectively, hDNAmad+ EC tumors display an immunologically cold phenotype. In contrast, significantly higher expression of inhibitory molecules including PD-L1, CTLA4, PDCD1, and TIGIT while diminished abundances of VTCN1 were observed in hDNAmad- tumors. Cancer immune cycle analyses further unraveled reduced recruitments of most immune cells coupled with fewer cell infiltrations and tumor killing. Immunoexclusion is associated with poor response to ICI therapy, and thus, the present findings are implicated in precision immunotherapy. It will be interesting to evaluate whether patients in the hDNAmad+ group are resistant to ICI therapy.

Proficient MMR, lower TMB, and stemness phenotype may provide explanations for immunoexclusion in hDNAmad+ EC tumors (7, 41, 49, 50), however, it remains incompletely understood whether other mechanisms are involved. More recently, aging- and cancer-related hypomethylation has been shown to induce reactivation of *HERV* gene transcription (34, 51, 52). The ERV-derived products are recognized as “non-self” by the host’s innate and adaptive immune system (34, 53). Importantly, transcribed viral double-strand RNAs (dsRNAs), RNA : DNA hybrid, and DNA or cDNA all trigger strong innate immune responses through the activation of pathogen recognition receptors (53). By doing so, the host immune system recognizes and treats cancer cells as virus-infected cells, the so-called “viral mimicry” effect (53). However, we did not observe consistent differences in HERV expression between hDNAmad+ and – tumors. We only included 7 HERVs for analyses and obtained results were insufficiently conclusive. Nevertheless, Miranda et al. showed that cancer stemness anti-correlated strongly with the exclusion of immune cell infiltration and one of the mechanisms was attributable to cancer cell-intrinsic HERV silencing (41). Given the robustly increased stem cell phenotype of hDNAmad+ tumors, HERV repression may occur in these tumors, which calls for further investigations including all known HERVs.

Hyperactive proliferation coupled with enhanced stemness is another feature of hDNAmad+ tumors. Intriguingly, we observed very low SCGB2A1 expression coupled with a higher frequency of PIK3CA alterations in this tumor group. SCGB2A1 was recently identified to act as an inhibitor of the PI3K kinase, and its inhibitory effect is as potent as PTEN, a well-defined negative regulator in the PI3K-AKT cascade (44). In addition, the inactivation of tumor suppressors/aging-drivers TP53, RB1, and CDKN2A while enhanced telomere maintenance also promotes the proliferation of hDNAmad+ tumor cells.

It has been shown that DNA methylation loss in aged tissues induces chromatin reorganization, which consequently inhibits the expression of protein-coding genes in these regions, such as those involved in stemness, invasion, and metastasis (34). As described above, hypomethylation may also trigger HERV reactivation, thereby provoking innate and adopted immune responses to suppress carcinogenesis (34, 51, 52). Thus, age-related hypomethylation functions as a barrier to malignant transformation and progression (22, 34). It is thus conceivable that hDNAmad+ EC tumors exhibit an aggressive phenotype. Intriguingly, robustly higher levels of DNMT3A and 3B were observed in these tumors and their increased expression may rescue global DNA hypomethylation to circumvent its tumor suppressive effect. On the other hand, EC patients with diabetes or hypertension exhibited a significantly lower incidence of hDNAmad+ tumors. Previous publications have shown a DNAmaa in patients with either diabetes or hypertension (16, 19). Likely, the existence of DNAmaa attenuates the development of DNAmad+ EC tumors. Taken together, the recovery of aging-like DNA hypomethylation may inhibit the progression of hDNAmad+ EC tumors.

In summary, by analyzing DNAm age in ECs, we identified a hDNAmad+ tumor subtype with an aggressive phenotype. This group of tumors exhibits very low expression of SCGB2A1 and increased frequency of PIK3CA alterations through which hyperactive proliferation and stemness are achieved. They are molecularly characterized by high CNAs and proficient dMMR while low TMB. Immunoexclusion is the featured microenvironments of hDNAmad+ tumors, suggesting a poor response to ICI therapy, which is implicated in EC precision immunotherapy. We further demonstrate that hDNAmad serves as an independent prognostic factor. Collectively, DNAm age deceleration may overcome tumor suppressive activities mediated by aging-related hypomethylation, and thus, the present findings are of importance both biologically and clinically.

## Data availability statement

The original contributions presented in the study are included in the article/**Supplementary Material**. Further inquiries can be directed to the corresponding authors.

## Author contributions

TL, JH, HY, and DX conceived and designed the study. JH, HY, YX, and TL performed bioinformatics analysis. TL and DX supervised the study. TL, JH, HY, and DX wrote and revised the manuscript. All authors contributed to the article and approved the submitted version.
